# Sero‐epidemiologic study of influenza A(H7N9) infection among exposed populations, China 2013‐2014

**DOI:** 10.1111/irv.12435

**Published:** 2017-01-21

**Authors:** Nijuan Xiang, Tian Bai, Kai Kang, Hui Yuan, Suizan Zhou, Ruiqi Ren, Xiuying Li, Jiabing Wu, Liquan Deng, Ge Zeng, Xianjun Wang, Shenghua Mao, Jian Shi, Rongbao Gao, Tao Chen, Sumei Zou, Dan Li, Fiona Havers, Marc‐Alain Widdowson, Carolyn M. Greene, Yanping Zhang, Daxin Ni, Xiaoqing Liu, Qun Li, Yuelong Shu

**Affiliations:** ^1^ Chinese Center for Disease Control and PreventionBeijingChina; ^2^ National Institute for Viral Disease Control and PreventionCollaboration Innovation Center for Diagnosis and Treatment of Infectious DiseasesChinese Center for Disease Control and PreventionKey Laboratory for Medical VirologyNational Health and Family Planning CommissionBeijingChina; ^3^ Henan Provincial Center for Disease Control and PreventionZhengzhouHenan ProvinceChina; ^4^ Jiangxi Provincial Center for Disease Control and PreventionNanchangJiangxi ProvinceChina; ^5^ United States Centers for Disease Control and PreventionAtlantaGAUSA; ^6^ Xinhui District Center for Disease Control and PreventionJiangmenGuangdong ProvinceChina; ^7^ Anhui Provincial Center for Disease Control and PreventionHefeiAnhui ProvinceChina; ^8^ Jilin Provincial Center for Disease Control and PreventionChangchunJilin ProvinceChina; ^9^ Hunan Provincial Center for Disease Control and PreventionChangshaHunan ProvinceChina; ^10^ Shandong Provincial Center for Disease Control and PreventionJinanShandong ProvinceChina; ^11^ Shanghai Municipal Center for Disease Control and PreventionShanghaiChina; ^12^ Hebei Provincial Center for Disease Control and PreventionShijiazhuangHebei ProvinceChina

**Keywords:** avian influenza, close contact, general population, H7N9 virus, poultry workers, serology

## Abstract

**Background:**

The first human infections of novel avian influenza A(H7N9) virus were identified in China in March 2013. Sentinel surveillance systems and contact tracing may not identify mild and asymptomatic human infections of influenza A(H7N9) virus.

**Objectives:**

We assessed the seroprevalence of antibodies to influenza A(H7N9) virus in three populations during the early stages of the epidemic.

**Patients/Methods:**

From March 2013 to May 2014, we collected sera from the general population, poultry workers, and contacts of confirmed infections in nine Chinese provinces reporting human A(H7N9) infections and, for contacts, second sera 2‐3 weeks later. We screened for A(H7N9) antibodies by advanced hemagglutination inhibition (HI) assay and tested sera with HI titers ≥20 by modified microneutralization (MN) assay. MN titers ≥20 or fourfold increases in paired sera were considered seropositive.

**Results:**

Among general population sera (n=1480), none were seropositive. Among poultry worker sera (n=1866), 28 had HI titers ≥20; two (0.11%, 95% CI: 0.02‐0.44) were positive by MN. Among 61 healthcare and 117 non‐healthcare contacts’ sera, five had HI titers ≥20, and all were negative by MN. There was no seroconversion among 131 paired sera.

**Conclusions:**

There was no evidence of widespread transmission of influenza A(H7N9) virus during March 2013 to May 2014, although A(H7N9) may have caused rare, previously unrecognized infections among poultry workers. Although the findings suggest that there were few undetected cases of influenza A(H7N9) early in the epidemic, it is important to continue monitoring transmission as virus and epidemic evolve.

## 1 Background

The first human infections with influenza A(H7N9) virus were identified in China in March 2013^1^. As of July 19, 2016, there were 793 influenza A(H7N9) virus infections in humans reported globally, including 319 fatalities. Among all H7N9 cases reported, mainland China reported 770, including 315 deaths, in 16 provinces (Anhui, Fujian, Guangdong, Guangxi, Guizhou, Hebei, Henan, Hubei, Hunan, Jiangsu, Jiangxi, Jilin, Liaoning, Shandong, Xinjiang, and Zhejiang) and three municipalities (Beijing, Tianjin, and Shanghai)^2^. The remaining 23 cases were all imported from mainland China and included 16 cases and three deaths reported in Hong Kong, four cases and one death reported in Taiwan, one case in Malaysia, and two cases in Canada. Most influenza A(H7N9) virus infections in humans have been associated with direct or indirect exposure to poultry, including visiting live poultry markets (LPMs)^3^, ^4^, ^5^ and farms^6^, ^7^.

The large majority of persons identified with influenza A(H7N9) virus infection presented with severe disease^8^ and approximately 40% died^2^. Persons with mild, atypical, and asymptomatic infections are far less likely to be tested for influenza A(H7N9) and will usually not seek health care. The detection of mild infections through sentinel surveillance systems^9^ and through the tracing of contacts of patients with confirmed infection^10^ suggests that an unknown number of mild and subclinical infections went undetected. Serologic studies are needed to identify subclinical influenza A(H7N9) human infections and better describe the full spectrum of influenza A(H7N9) human infection. Although several serologic studies of influenza A(H7N9) have been published, they have been limited to single provinces^11^, ^12^, ^13^, ^14^ and have used either less sensitive serologic assays^11^ or lacked confirmation by microneutralization testing^11^, ^13^, ^14^, which improves specificity of serologic testing results^15^.

In this study, we examined the seroprevalence of antibodies to influenza A(H7N9) in three populations: the general population, poultry workers, and close contacts of persons with influenza A(H7N9) virus infection in affected provinces using specific serologic methods.

## 2 Patients and Methods

### 2.1 General population in two provinces

In the early stages following the identification of influenza A(H7N9) virus, we recruited members of the general population in two provinces, Jiangxi and Henan, where provincial governments expressed support for the study. Villages or counties with at least one reported human case of influenza A(H7N9) virus infection and the immediately adjacent neighboring villages and counties were eligible for enrollment. Village and county selection was based on willingness of the local governments to participate in the study. In Jiangxi Province, the first human influenza A(H7N9) virus infection was reported on April 24, 2013. We selected two villages with influenza A(H7N9) cases and seven neighboring villages, and collected data from May 21 to June 2, 2013. In Henan Province, the first human infection with influenza A(H7N9) virus was reported in one county on April 11, 2013. We selected two affected counties and 13 neighboring counties and conducted the study from April 21 to 27, 2013.

All residents in selected affected and adjacent villages were eligible to participate in this study. Residence was defined as the place where a person had spent the majority of nights in the past 3 months. We interviewed all residents who were at home during our visit to the selected villages. Our target sample size was 1000 participants from both provinces.

### 2.2 Poultry workers in six provinces

From April 2013 to May 2014, we collected data from poultry workers in six provinces: Jiangxi, Hunan, Anhui, Henan, Jilin, and Guangdong. Villages or counties where at least 1 month had passed since the first confirmed human case of influenza A(H7N9) virus had been reported and the adjacent neighboring villages and counties were eligible for inclusion in the study. Selection was based on willingness of the local governments to participate. In selected sites, we focused on two different types of work sites: live poultry markets (LPMs) (including wholesale markets) and farms (commercial farms or households raising small‐scale poultry).

All staff who earned at least 50% of their income from poultry work at selected markets and farms were eligible for this study. Workers from live poultry and wholesale market locations included sellers, butchers, cleaners, and transporters. Workers from farms included those from commercial farms or small‐scale farmers who raised poultry in their households for commercial sale. We enrolled all poultry industry workers from selected sites who consented to participate.

### 2.3 Close contacts in four provinces and one city

We selected all provinces with at least one influenza A(H7N9)‐confirmed case reported before March 2014 where the local CDCs agreed to participate in the study. We collected data from contacts of the following human cases: one case reported on March 31, 2013, in Shanghai; three cases reported during March 25 to April 15, 2013, in Anhui; one case reported on April 23, 2013, in Shandong; one case reported on July 20, 2013, in Hebei; and one case reported on February 22, 2014, in Jilin.

All healthcare contacts and non‐healthcare contacts of confirmed influenza A(H7N9) cases who were willing to participate were eligible. A healthcare contact was defined as one who provided direct medical care to an influenza A(H7N9) case, either before or after confirmation of diagnosis, and who did not use standardized personal protective equipment (PPE) protection as defined by national guidelines^16^. A non‐healthcare contact was defined as a family member who lived with or cared for the patient, as well as other persons who had close contact with the patient during the illness but prior to isolation and without PPE.

### 2.4 Data collection

Each study participant ≥18 years of age and guardians of participants <18 years of age completed one questionnaire, designed to collect information on demographic characteristics, health habits, and information on exposure to birds in the household, LPMs and other places (such as live poultry farms, wetland, and parks) in the preceding 3 months, respiratory symptoms in the preceding 3 weeks, and contact with persons with fever and respiratory illness in the preceding month.

### 2.5 Specimen collection

We collected a blood sample from each participant using venous vacuum blood collection tubes (Becton Dickinson Medical Devices (BD) Vacutainer SST). When possible, we collected paired blood samples from close contacts. We collected the first blood sample 7 days after their exposure to the confirmed case, and the second blood sample 2‐3 weeks later. The sera were temporarily stored at 4°C; after separation, sera were stored at −20°C in local public health laboratories. After the field investigation was completed, sera were sent to the National Influenza Center of China CDC.

### 2.6 Laboratory testing

We used a modified HI assay with horse red blood cells (RBCs) to screen for antibody response to influenza A(H7N9) virus according to the standard protocol endorsed by the World Health Organization (WHO)^17^. The influenza A(H7N9) antigen used in the HI assay was A/Anhui/1/2013, propagated in specific pathogen‐free (SPF) embryonated chicken eggs and inactivated with 1‰ β‐propiolactone (BPL). A positive serum control (ferret antiserum against A/Anhui/1/2013) and a negative serum control (stock sera from healthy populations collected in 2009 prior to the emergence of influenza A(H7N9) virus) were included in each assay. We modified the order of treatment by conducting hemadsorption before applying receptor‐destroying enzyme (RDE) treatment on serum samples. Samples with an HI titer ≥20 were tested using the same virus (A/Anhui/1/2013) by neutralization antibody detection by a modified microneutralization (MN) assay for confirmation^15^. Seropositivity was defined as a MN titer ≥20 or a fourfold titer increase in paired samples^15^.

### 2.7 Data analysis

Epidata3.0 was used for parallel data entry and checking. spss18.0 (SPSS Inc., Chicago, IL, USA) was used to conduct frequency analyses.

## 3 Results

### 3.1 General population

**Figure 1:**
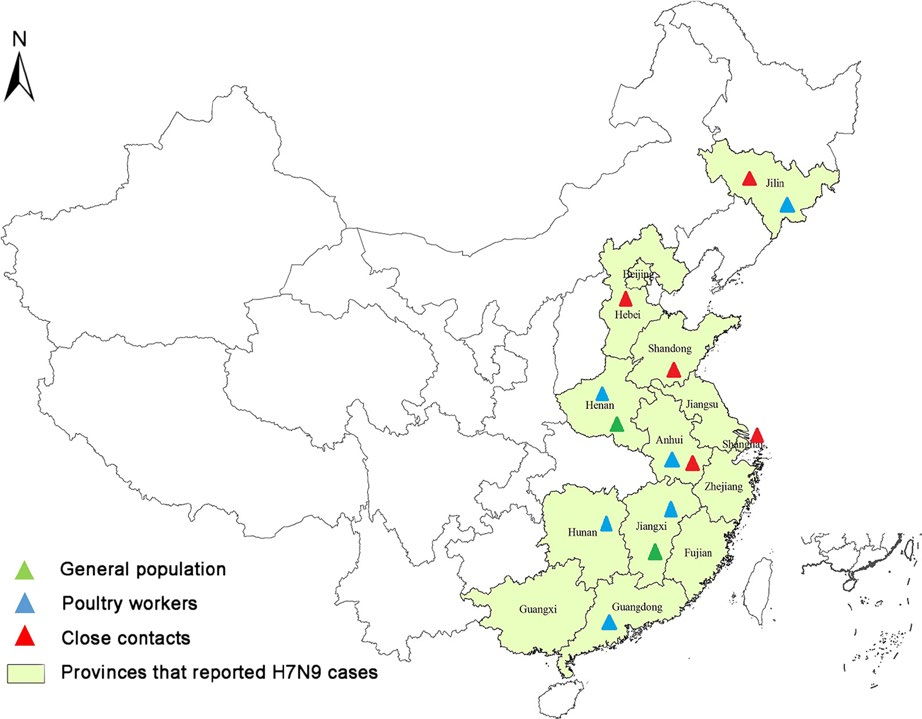
Sites for influenza A(H7N9) serologic survey among the general population, poultry workers and close contacts in mainland China, March 2013 to May 2014. This figure shows the geographic distribution of the different risk populations studied. The colored triangles represent the different populations included by province. The area shaded in light green represents provinces with reported A(H7N9) cases in mainland China during the study period

We enrolled and collected blood from a total of 1480 people from the general population; 1054 subjects were from Jiangxi Province and 426 subjects from Henan Province (Figure 1). The median age of these participants was 49 years, ranging from 6 months to 93 years; 42% (627) were male. Participants from every age group were enrolled in this study (Table 1).

**Table 1:** Characteristics of enrollees in the general population in Henan (15 counties) and Jiangxi (nine villages), April to June 2013

Age category (years)	Henan (first case confirmed on April 14, 2013)			Jiangxi (first case confirmed on April 26, 2013)			Total, n (%)
	Male, n (%)	Female, n (%)	Total, n (%)	Male, n (%)	Female, n (%)	Total, n (%)	
0‐5	1 (0.5)	0 (0.0)	1 (0.2)	14 (3.2)	8 (1.3)	22 (2.1)	23 (1.6)
6‐17	7 (3.6)	9 (3.9)	16 (3.8)	79 (18.2)	58 (9.3)	137 (13.0)	153 (10.3)
18‐59	158 (81.4)	196 (84.5)	354 (83.1)	215 (49.7)	372 (59.9)	587 (55.7)	941 (63.6)
≥60	28 (14.4)	27 (11.6)	55 (12.9)	125 (28.9)	183 (29.5)	308 (29.2)	363 (24.5)
Total	194 (45.5)	232 (54.5)	426 (100.0)	433 (41.1)	621 (58.9)	1054 (100.0)	1480 (100.0)

Among all samples, none had an HI titer to influenza A(H7N9) ≥20, which allowed us to exclude with 95% confidence a true seropositivity in this population of more than 0.32%.

### 3.2 Poultry workers

We enrolled and collected blood from 1866 poultry workers from six provinces (Figure 1). The median age of these participants was 44 years (interquartile range: 15‐51 years). Of them, 1527 (82%) worked in LPMs and 337 (18%) in commercial or small‐scale farms. All 1146 specimens from Henan (915), Jiangxi (25), Hunan (33), and Jilin (173) had an HI antibody titer <20, as did all, but 3 (2%) of 143 specimens collected in Anhui Province. These three samples, all with HI antibody titers of 40, were negative by MN testing (Table 2). In Guangdong, 25 (4%) of 577 specimens had an HI antibody titer ≥20, and two (0.3%) were positive by MN titer. Both positive samples were from the specimens collected April to May 2014 from Xinhui Prefecture City. Therefore, the seropositivity among all poultry workers tested was 2 of 1866 (0.11% (95% CI: 0.02‐0.44)).

**Table 2:** Hemagglutination inhibition (HI) and microneutralization (MN) antibody titers against influenza A(H7N9) among poultry workers in six provinces, April 2013 to May 2014

Provinces	Date first case confirmed	Time of collection	No. of specimens	HI titer		MN titer a	
				Neg, N	Pos, N (titer)	Neg, N	Pos, N (titer)
Henan	April 14, 2013	April 2013	915	915			
Jiangxi	April 26, 2013	May 2013	25	25			
Hunan	April 24, 2013	May 2013	33	33			
Anhui	March 30, 2013	July 2013	143	140	3 (40)	3	
Guangdong	August 10, 2013	August 2013	14	14			
		January 2014	86	75	9 (20)	9	
					2 (40)	2	
		April to May 2014	477	463	9 (20)	9	
					4 (40)	3	1 (40)
					1 (80)		1 (40)
Jilin	Feb 21, 2014	April to May 2014	173	173			
Total		April 2013 to May 2014	1866				

a MN test was conducted for those with HI titer ≥20 only.

Both MN‐positive samples were from female workers: One worked in a wholesale market and the other in a LPM. Neither reported any respiratory illness in the 3 weeks prior to specimen collection, and neither had been hospitalized due to respiratory illness during the preceding year. Additional demographic characteristics and exposure information are shown in Table 3. Neither market had been tested for influenza A(H7N9) avian influenza virus. However, the wholesale market provided poultry to another market with positive influenza A(H7N9) environmental samples^18^.

**Table 3:** Poultry workers who tested positive by microneutralization (MN) assay for influenza A(H7N9), Guangdong, April to May 2014

Age (years)	Gender	Workplace	Job type	Febrile respiratory illness a	Exposure	Routine protection	Date of specimen collection	HI titer	MN titer
50	Female	Wholesale market	Seller	No	Direct contact with live poultry	Gloves, mask, rubber overshoes	May 12 2014	80	40
48	Female	Live poultry market	Butcher	No	Slaughter poultry	Gloves, rubber overshoes	April 29 2014	40	40

a Febrile respiratory illness in the 3 weeks preceding specimen collection.

### 3.3 Close contacts of infected patients

We enrolled and collected blood from 61 healthcare contacts and 117 non‐healthcare contacts of persons with confirmed influenza A(H7N9) virus infection from five provinces (Figure 1). The median ages were 31 years (interquartile range: 20‐43 years) among healthcare contacts and 33 years (interquartile range: 28‐54 years) among non‐healthcare contacts. Paired samples were collected from 47 (77%) of the healthcare contacts and from 84 (72%) of the non‐healthcare contacts.

Among all samples collected, one sample from a healthcare contact in Shanghai had an HI titer of 40 and four samples from non‐healthcare contacts in Shandong had HI titers of 20 (1), 40 (2), and 80 (1), respectively. All five specimens tested negative for influenza A(H7N9) MN antibody (<1:10) (Table 4). There was no seroconversion within the 131 paired blood samples. We could exclude a true seropositivity of more than 1.53% in this population (upper limit of the 95% CI).

**Table 4:** Hemagglutination inhibition (HI) and microneutralization (MN) antibody titers against influenza A(H7N9) among close contacts, March 2013 to February 2014

	Provinces	Date first case confirmed	No. of blood sample	Time of collection	HI titer	MN titer c,
Healthcare contacts	Shanghai	March 30, 2013	14 single^a^	March 2013	1 sample: 40All others <20	All <10
	Anhui	March 30, 2013	16 pair b	April 2013	All <20	
	Jilin	Feb 21, 2014	31 pair	February 2014	All <20	
Non‐healthcare contacts	Shandong	April 23, 2013	10 pair	April 2013	1 sample: 202 sample: 401 sample: 8016 sample<20	All <10
	Anhui	March 30, 2013	54 pair33 single	April 2013	All <20	
	Hebei	July 20, 2013	5 pair	July 2013	All <20	
	Jilin	Feb 21, 2014	15 pair	February 2014	All <20	
Total			309 samples			

a Single blood samples were collected from close contacts during convalescent period of the index patient they were exposed to.

b Pair blood samples were collected from close contacts during both acute and convalescent period of the index patient they were exposed to.

c MN test was conducted for those sera with HI titer ≥20 only.

## 4 Discussion

This study used hemagglutination and neutralization assays to examine influenza A(H7N9) virus transmission to the general population, poultry workers, and close contacts of laboratory‐confirmed influenza A(H7N9) cases, including healthcare workers, in areas of influenza A(H7N9) virus circulation from March 2013 to May 2014 in multiple provinces in China. Among the general population sample and among close contacts, we found no subjects who were seropositive for influenza A(H7N9) by MN testing. Among workers in the poultry industry, two of 1866 had a positive MN result. These findings suggest that in the first year following the detection of this emerging virus, influenza A(H7N9) transmission in various populations—including those with high exposure risks—was generally low.

The low prevalence of confirmed seropositive subjects in this study might have been anticipated because the influenza A(H7N9) virus lacks efficient binding to human receptors^19^. We used the same testing method to examine 1544 stored samples collected in December 2012 among poultry workers in the Yangtze River Delta region^20^. Results of the banked sample testing showed no evidence of human infection with influenza A(H7N9) prior to the identification of the virus in March 2013, which indicated that transmission likely began shortly before this study was initiated in April of that year. Among samples with elevated HI titers, we observed relatively lower corresponding MN titers. This was consistent with previous experience in which the neutralizing antibody response among confirmed influenza A(H7N9) cases was relatively weak compared with the neutralizing antibody response against the 2009 pandemic A(H1N1) and avian influenza A(H5N1) virus using a similar MN assay^21^.

In the general population sample from two provinces examined in this study, we found no indication of infection, consistent with the findings of studies conducted in Zhejiang Province^11^ and Shenzhen, Guangdong province^13^ in 2013. Our study also found that among close contacts of infected patients, although several had an elevated HI titer, MN confirmation showed no evidence of infection; this is consistent with the results of a serologic study conducted in Guangdong in July 2013, which included close contacts^12^.

Although we identified two seropositive cases in poultry workers, the overall seropositivity in this study was much lower than that reported in previous serologic studies conducted in a similar time period^11^, ^13^, ^14^. Compared with studies that did not perform MN testing, the proportion of elevated HI titers in our study (0.1% poultry workers with HI titer ≥20) was also much lower. For example, one study among poultry workers in Shenzhen found 7.2%‐14.9% with HI titers ≥160^13^. Another in Guangzhou found 1.6% poultry workers with HI titers ≥40^14^, and a study in Zhejiang Province reported 6.3% poultry workers with HI titers ≥80^7^. Although our study found a lower seropositive rate among poultry workers in Guangdong than prior studies conducted in this province^12^, ^13^, ^14^, our study showed a higher seroprevalence among poultry workers in Guangdong compared with the poultry workers from the other five provinces included in the study. Indeed, both of the two poultry workers with positive H7N9 neutralization antibodies in this study were from Guangdong. This finding is not surprising, as it is likely that subclinical infection of H7N9 virus in poultry workers is highest in the provinces with greatest H7N9 virus circulation. At the time of this study, the province with the greatest number of H7N9 cases reported among our study provinces was Guangdong^22^.

Although technical differences between serologic assays limit comparison between studies, the serologic studies conducted during the early stage of the influenza A(H7N9) outbreak all suggest that during the first year of the influenza A(H7N9) outbreak, healthcare workers and other close contacts of laboratory‐confirmed influenza A(H7N9) cases were at low risk of infection. Further, there was little sustained, widespread transmission of influenza A(H7N9) virus in the general population. Conducting serologic studies at the early stage of an outbreak with an emerging virus is particularly important for a novel avian influenza virus like influenza A(H7N9). Although the findings from these early serologic studies are reassuring and suggest that the number of undetected cases of influenza A(H7N9) during the first months of the outbreak was low, it will be important to continue monitoring transmission closely as the virus and the epidemic evolve.

This study has several limitations. Initiated as part of the public health response in the very early stages of the influenza A(H7N9) outbreak, when little was known about the extent of spread of the disease, this study initially lacked rigorous study design and sampling methods. For example, we enrolled the entire general population sample from Jiangxi and Henan provinces. The number of participants enrolled in Henan was less than our target, likely due to low awareness of A(H7N9) at the beginning of the outbreak contributing to a high refusal to participation rate. Further, as both Jiangxi and Henan provinces had lower numbers of reported cases compared with other areas^22^, our study may have underestimated the real infection rate of influenza A(H7N9) in the general population. In addition, our study only included close contacts of the early subset of all human influenza A(H7N9) cases. Although we found no evidence of infection among close contacts in our study, several clusters among close contacts of later cases were detected, indicating that the first generation of human‐to‐human transmission likely occurred on multiple occasions, although we did not detect evidence of this in our sample^23^, ^24^. With respect to our serologic study of poultry workers, we do not know what proportion of workers in our study were exposed to H7N9 in their workplaces, as several LPMs, including those where the two poultry workers with seropositive samples worked, had not been tested for influenza A(H7N9) avian influenza virus. This may have led to a lower seroprevalence among poultry workers than we would have found had we only enrolled workers from LPMs with positive influenza A (H7N9) environmental samples. Finally, it is important to note that serologic testing may not detect all infections due to waning or lack of antibody response to influenza A(H7N9) infection, especially mild infection, and thus, our study may underestimate influenza A(H7N9) infection rates during the early stages of the outbreak^15^, ^21^.

In summary, our study suggests that there was minimal transmission of influenza A(H7N9) virus during March 2013 to April 2014 among the general population, poultry workers, and close contacts of confirmed cases. However, because influenza A(H7N9) was first detected and this study was conducted, the geographic spread of the virus has increased and human cases continue to occur. Serologic studies play an important role in assessing the extent of influenza A(H7N9) transmission in humans, and ongoing assessment and vigilant monitoring of the viral evolution and epidemiology of this emerging pathogen is crucial.

## Conflict of Interest

We declare that we have no conflicts of interest.

## Disclaimer

The opinions expressed by authors contributing to this journal do not necessarily reflect the opinions of the Centers for Disease Control and Prevention or the institutions with which the authors are affiliated.
